# Perioperative Use of Herbal, Complementary, and Over the Counter Medicines in Plastic Surgery Patients

**Published:** 2011-05-19

**Authors:** Declan Collins, Steve Oakey, Venkat Ramakrishnan

**Affiliations:** St Andrew's Centre for Burns and Plastic Surgery, Court Road, Broomfield, Chelmsford, Essex, United Kingdom

## Abstract

**Objective:** Over the last 50 years, there has been a surge of interest by both the public and medical practitioners in therapies and disciplines that are not considered part of mainstream medical care. The title given to these is complementary and alternative medicine. Of all these branches, our interest is the increasing use of herbal medicines, traditional medicines (such as Chinese or Indian), homeopathy and “dietary supplements,” and the influence they may have on our practice. Our objective was to examine the prevalence and reasons for use of complementary and alternative medicines, the current regulations, and proposed policy changes affecting the licensing of these products. In addition, we highlight some of the problems that have been experienced with herbal and traditional medicines. **Methods:** A prospective analysis of herbal and over the counter medicines used by elective plastic surgery patients. **Results:** Of 100 elective plastic surgery patients undergoing procedures at St Andrew's Centre for Burns and Plastic Surgery, 44% of patients were taking a dietary supplement, herbal, or homeopathic remedy. In none of the patients was this documented in the notes by either the surgeon or anesthetist. **Conclusions:** We recommend that clear documentation of the use of nonprescribed medicines becomes part of standard practice and, furthermore, that patients stop all such medications 2 weeks prior to surgery until the efficacy, interactions, and safety profiles are clearly established.

## BACKGROUND

The first synthetic chemical drug was aspirin, manufactured by Bayer in Germany, and put on the market in 1899. Prior to this, western medicine has been similar to that practiced in all other parts of the world (such as Chinese or Ayurvedic) in that it was mainly based on the use of plants and herbs, animal preparations with possibly the addition of metal compounds.^1^

Since 1899, the number and use of synthetic drugs has increased hugely making it the mainstay treatment for the majority of medical practitioners. Concurrently with great pharmaceutical advances have been improvements in diagnosis and surgical innovations of which plastic surgery has often been at the forefront.

For a variety of reasons, over the last 50 years, however, there has been a surge of interest by both the public and medical practitioners in therapies and disciplines that are not considered part of mainstream medical care. The title given to these is complementary and alternative medicine (CAM)

This title encompasses disciplines as wide ranging as aromatherapy to acupuncture and homeopathy to osteopathy. It is only recently that some UK medical schools have made the study of CAM, a part of the curriculum.

Of all these branches of CAM, our interest is the increasing use of herbal medicines, traditional medicines (such as Chinese or Indian), homeopathy, and “dietary supplements” and the influence they may have on our practice.

## PREVALENCE OF CAM USE

Figures vary in studies over the world but all tend to show an increase in CAM. A telephone survey conducted by British Broadcasting Corporation in 1999 showed 20% of people had used some form of CAM in the preceding year, 34% of which had taken herbal remedy.^2^

A larger postal survey of CAM use in England found 8.6% of respondents had bought an over the counter homeopathic remedy and 19.8% had bought an over the counter herbal remedy.^3^

Around the time of these studies, sales of complementary medicines (defined here as herbal, homeopathic, and aromatherapy essential oils) totaled £93 million in 1998^1^ and had increased to £130 million in 2002. They now total an estimated £213 million per annum on the basis of work by market researchers Mintel.^4^ Sales of herbal remedies alone were £43.7 million in 1997, £65 million in 2000, and forecasted to be £117 million in 2007.^4^

The UK picture echoes a similar scene in the United States where herbal medicine use has increased from 2.5% in 1990 to 12.1% in 1997. Expenditure on alternative therapies was estimated to be $21.0 billion in 1997^5^ that rose to $27.0 billion by 2005, with one-third of the American public using CAMs.^47^

## THE REASONS FOR USING CAMs

The hypotheses for why patients may choose CAMs are multiple. For some patients, it is a way of empowerment and allowing greater control over their health care decisions.^6^ For others, it may be a distrust or dissatisfaction with conventional medicine, physicians, or hospitals^7^; although, it must be noted that many patients use CAMs in conjunction with conventional treatments^8^ and often for health maintenance rather than to treat a specific ailment. For many, however, it is due to a successful resolution or reduction of symptoms or as part of the patient's life style or philosophy of life.^9^ For others, it may be through media exposure of celebrities using CAMs.^13^ When given a range of statements questioning why they used herbal or homeopathic remedies, 43% of respondents cited recommendations by family or friends while only 18% claimed it was because of concern of the side effects of standard medications.^4^

A US postal survey looked at why people access CAM and predictors for CAM use. In addition to those factors noted earlier, chronic health problems (ie, anxiety, pain, back problems) were also found to be associated with CAM use.^9^,^11^

Significant predictors for CAM use have also found to be of high-educational status,^7^,^11^ overall health status,^9^,^10^ female sex,^6^,^10^ and higher than average social class.^6^ There is also a geographical component to CAM use in the United Kingdom, with a higher proportion of residents in London using herbal or homeopathic remedies compared with other parts of the United Kingdom.^4^

## REGULATION AND POLICY

A key concern in recent years is the level of regulation that herbal and traditional remedies have.

Article 1 of European Council Directive 2004/27/EC as amended defines a “medicinal product” as

(*a*) Any substance or combination of substances presented as having properties for treating or preventing disease in human beings;

(*b*) Any substance or combination of substances, which may be used in or administered to human beings either with a view to restoring, correcting or modifying physiological functions, by exerting a pharmacological, immunological or metabolic action, or to making a medical diagnosis.^12^

This in effect means that any product supplied with therapeutic intent (eg, by having a claim on the label) is a medical product. Therefore, all products that make claims to treat diseases are considered medicines and subject to regulation by European Union Directives and the Medicine Act. All products that make a medicinal claim require a product license (also referred to as marketing authorization by the Medical Control Agency) to ensure a certain standard of quality and safety.

Some herbs are currently exempted from licensing if they meet certain conditions (Section 12 Medicines Act, 1968), which allows sales of herbal remedies (without medicinal claims) if the manufacturing process is simple (ie, consists of drying or crushing) or if supplied to individual patients following a face-to-face consultation.

Because of differing regulations and uncertainty in the classifications of herbal remedies in the EU member states, a new scheme was introduced in October 2005—the Traditional Herbal Medicines Registration Scheme. This is designed to protect public health by requiring specific standards of safety and quality for traditional herbal medicines. This scheme is required by the European Directive on Traditional Herbal Medicinal Products (2004/24/EC).

This scheme has 2 methods of registration. First, medical use registration (which is similar to the previously used product licence), which means they have achieved marketing authorization, this will include all those herbs that have been audited to pharmaceutical standards and can make claims regarding use and efficacy. Second, traditional use registration for those herbs that do not fulfil the criteria of the previous category but are manufactured on licensed premises and can supply evidence of “well-established medical use,” which is defined as medical use in the European Union for 30 years (products from outside the European Union must prove at least 15 years within Europe and 15 years outside Europe). By April 2011, all manufactured herbal medicines sold over the counter will be required to have either a traditional herbal registration or a product license.^48^

Because of the difficulty in getting new herbal remedies market authorization many herbal products, especially from China and India are not licensed as medicines and are, therefore, supplied to the public without specific regulations. This means that standards of preparation and content can vary greatly between products bought over the counter.

## POTENTIAL PROBLEMS

There are several areas of concern with the increased use of ingested complementary or alternative medicines. The first area, mentioned earlier, which is currently being addressed, is the regulation ensuring conditions of use and standards of efficacy and safety.

Second is the possibility of side effects from use of nonprescribed medication. A common misperception is that herbal remedies are somehow more natural and, therefore, safer. However, side effects of herbal remedies are more frequently being reported whether through increased usage and awareness or whether the fact that easier routes for reporting side effects are now available is unclear.

A number of well-recognized herbal remedies have been found to be associated with serious side effects or toxicity.

A recently reported example of a commonly use herbal remedy with serious side effects is Kava. Kava-kava is a perennial shrub native to some islands of the South Pacific from which a psychoactive drink can be made. In Pacific Island societies, it is sometimes used for ceremonial as well as medicinal purposes.^14^ In Europe, its extracts have been used as an anxiolytic. In January 2003, the government prohibited Kava-kava in unlicensed herbal medicines following reports worldwide of idiosyncratic liver damage associated with the consumption of Kava-kava. This was on the basis of advice from the committee on the safety of medicines that Kava-kava posed a rare but serious risk to public health. There have been a number case reports in the literature.^15^,^16^

Other herbal remedies have been associated with hepatotoxic events^15^, many of them herbs used in Chinese traditional medicines.

A third possible problem identified is the interaction of herbal remedies with concurrently prescribed medicine. An example is St John's Wort (*Hypericum perforatum*), which has been used to treat depression. Although a previously popular remedy available over the counter, it was found that *Hypericum* is a potent inducer of several cytochrome P450 enzymes^15^,^17^^-^19 and has been associated with a number of cases of drug interactions.^20^^-^22

A further point of concern is the possible contamination of herbal remedies. The contamination may be related to the environment in which the herbs or plants are grown or may occur during the processing of the substances in question. There have been concerns that without careful monitoring high-organochlorine pesticides levels may contaminate herbal medicines, especially those in which the stems, roots, or tubers are used.^23^,^24^

Contamination by heavy metals,^25^ which is particularly prevalent in Chinese traditional medicines^26^^-^28 has also been found. There are cases of poisoning with lead^29^^-^31,33, arsenic^29^, thallium^32^, cadmium,^33^,^34^ and mercury.^33^ A Taiwanese study looked at a randomly selected group of 2803 patients and screened them all for elevated blood lead levels. A history of using traditional Chinese medicines was shown to a significant risk factor for raised blood lead levels,^35^ which may suggest the problem may be more widespread than the case reports indicate.

A final potential problem of CAMs is that of adulteration. There have been a number of case reports and studies into adulteration of herbal remedies, with the adulterants often posing a significant risk.^14^,^36^ A study in Taiwan found that 24% of all samples of traditional Chinese medicines contained adulterants, half of which contained 2 or more adulterants.^37^ In addition to this, adulteration has also been found in homeopathic treatments.^40^

## PATIENT SURVEY

To examine our own patient group a survey of 100-elective plastic surgery patients was conducted. All patients were preclerked prior to the day of operation. Their notes were checked to see whether any herbal/Chinese/homeopathic or over the counter medicines had been documented by either the anesthetist or the doctor that had performed the patient clerking. The patients were then asked whether they were currently taking any nonprescribed remedies. A list of 13 commonly used over the counter remedies were used to guide the patients. Any treatments not listed were also noted. The age and sex of the patients was recorded and vitamin and mineral supplements were also noted

Results were tabulated and differences in the use of herbal or homeopathic treatments compared to the age and sex of the patient were tested using χ^2^ analysis. A *P* value of less than .05 was considered statistically significant.

## RESULTS

Of 100 elective plastic surgery patients undergoing procedures at St Andrew's Centre for Burns and Plastic Surgery, Chelmsford, 44% of patients were taking a dietary supplement, herbal or homeopathic remedy of which 17% were taking a herbal or homeopathic remedy see Table 1.

Male-female ratio was 22:78. The average age of female patients was 56.96 years, range: 16 to 95 years; average age of male patients was 54.54 years, range: 18 to 104 years (see Table 2). Some patients took more than 1 type of over-the-counter medicine with one patient taking 8 supplementary nonprescribed tablets and a topical application of a further remedy.

A wide variety of herbal medicines are being taken by the patients, as evidenced by 16 different types of herbal remedies being ingested by the 15 patients (Table 3). Twelve different types of vitamin or dietary supplement were used, the most common supplements being cod liver oil tablets and multivitamins.

Fourteen of the 15 patients taking herbal remedies were female (*P* < .05) with one male and one female patient taking a homeopathic remedy. Interestingly of the 2 patients taking homeopathic, neither of these patients knew what the name or content of the treatment they were ingesting.

Of all patients investigated, there was no record in the surgical preoperative clerking or anesthetic records of CAM use.

## DISCUSSION

The use of complementary and alternative medicines is growing in the United Kingdom. The use of herbal, homeopathic, and traditional remedies such as Chinese and Indian remedies form part of this market and may have a profound impact on patient outcomes perioperatively.

Our survey shows that a substantial number of patients are taking food supplements, homeopathic, or herbal remedies (44%)—a figure that endorses the growing use of CAMs described in the literature. Evidence shows that this market is growing in the United Kingdom and is following the trend seen in the United States over the last 2 decades. In addition, this market is also projected to continue growing.

Of the patients using CAMs a large proportion are taking herbal remedies, the group that potentially is in the most danger are those taking them perioperatively. Again our findings echo those of other larger studies both in the United Kingdom and in the United States although a comprehensive study in the United Kingdom in 2002 found a smaller (4.8%) proportion taking herbal remedies.^38^ However, this level of herbal use is in line with other studies.^3^ The typical patient taking a herbal remedy is female, middle aged, and often from a higher-income household, a demographic that forms a large proportion of elective plastic surgery patients especially in the private sector.

Although the regulation of herbal and traditional medicines is changing, there are concerns that the increased difficulty in getting a new drug licensed means more compounds will not be marketed as medicines and, therefore, be supplied without specific regulations. Therefore, surgeons and anesthetists should be particularly aware of potential interactions and side effects of the most commonly used CAMs. Two other useful studies have looked at the most frequently used CAMs in plastic surgery patients as well as potential interactions and complications.^41^,^42^ Even after the regulations come into effect awareness should still be maintained as the source of the treatment cannot always be verified (especially if bought over the internet or brought from a country with less stringent regulation). A study of 443 Web sites found that a significant proportion made unverifiable or illegal claims regarding the herbal remedies or nutritional supplements advertised.^43^

Our study shows that both surgeons and anesthetists poorly record the documentation of the use of CAMs by patients, as we did not find their use documented in either the surgical preoperative patient clerking or in the anesthetic records of a single patient that was investigated. We could not, however, establish in all cases whether the patients had been asked and either CAM use was not recorded or not thought to be sufficiently important to document. A survey of anesthetic practice in the United Kingdom highlighted this problem by showing that 90% of the anesthetists questioned stated that they seldom or never asked patients about herbal medicine usage.^44^ A further problem is that the information is unlikely to be volunteered by the patient. Around 70% of patients do not disclose the fact that they are using a CAM to their doctor.^45^ This study listed a variety of reasons including “It wasn't important for the doctor to know,” “The doctor never asked,” “It was none of the doctor's business” with some respondents thinking their doctor would disapprove of or discourage CAM use.

We suggest that the questioning of all patients and clear documentation regarding the use or not of CAMs and over the counter drugs becomes part of standard practice. A useful mnemonic has been made specifically for this purpose.^39^

Suggestions have been made for the time period for stopping certain herbal remedies prior to surgery,^46^ however, until the regulations are enforced we follow the American Society of Anaesthesiologists recommendations that patients stop all such medications 2 weeks prior to surgery until the efficacy, interactions and safety profiles are clearly established.

## Figures and Tables

**Table 1:** The percentage of patients taking unprescribed over the counter remedies

Type of supplement taken	Percentage of patients
Dietary supplement	37
Herbal supplement	15
Homeopathic remedy	2

**Table 2:** Age ranges of patients and over the counter remedies taken

Age range, y	No. of patients	Percent taking herbal remedies	Percent taking vitamin supplements	Percent taking dietary supplements
15-19	5	0	0	0
20-24	5	40	0	0
25-34	8	25	25	0
35-44	10	20	50	20
45-54	12	17	33	25
55-64	23	26	22	35
65+	37	3	16	24

**Table 3:** Individual herbal remedies and dietary/food supplements ingested

Remedy		n	%
	A*		
Arnica		1	4.8
Blueberry tablet with marigold		1	4.8
Aloe vera tablets		1	4.8
Black cohosh		1	4.8
Echinacea		2	9.6
Evening primrose oil		4	19.0
Garlic		2	9.6
Green tea tablets		1	4.8
Horse chestnut		1	4.8
Milk thistle		1	4.8
Periwinkle		1	4.8
Sage leaf capsules		1	4.8
Soya isoflavan		1	4.8
Star flower oil		1	4.8
Symphytum		1	4.8
Whole birch		1	4.8
	**B**†		
Multivitamins		15	25.0
Vitamin B		2	3.4
Vitamin C		7	12.0
Vitamin E		2	3.4
Calcium		1	1.7
Magnesium		1	1.7
Iron		1	1.7
Zinc		2	3.4
Cod liver oil		16	27.0
Glucosamine		7	12.0
Omega III		4	6.8
Lutein tablets		1	1.7

*Herbal remedies taken by 15 patients (n = 21).

†Vitamins and dietary supplements taken by 37 patients (n = 59).
